# Analyzed and Simulated Prediction of Emission Characteristics of Construction Dust Particles under Multiple Pollution Sources

**DOI:** 10.1155/2022/7349001

**Published:** 2022-07-07

**Authors:** Wei Liu, Xiaohui Huang, Huapeng Chen, Luyao Han

**Affiliations:** ^1^School of Civil Engineering and Architecture, East China Jiaotong University, Nanchang 330013, China; ^2^Civil Engineering Department of Engineering Science University of Greenwich, London, UK

## Abstract

Dust pollution in construction sites is an invisible hazard that is often ignored as a nuisance. Regulatory and engineering control methods are predominantly used for its mitigation. To control dust, dust-generating activities and their magnitudes need to be established. While researchers have comprehensively studied dust emissions of construction work, prediction of dust concentrations based on work phases and climatic conditions is still lacking. To overcome the above knowledge gap, this article selected two construction stages of a project to monitor dust generation using the HXF-35 dust sampler. Based on the collected data, dust emission characteristics of these two stages are studied, and dust emission characteristics under multiple pollution sources are analyzed. Based on the results, a BP neural network model is built to perform simulations of dust emission concentrations in different work areas and predict construction dust concentrations under different conditions. Except few, the majority of the work areas monitored have exceeded the allowable upper limit of TSP concentration stipulated by relevant standards. In addition, dust emission differences of work areas are pronounced. The results verified that the BP neural network dust concentration prediction model is feasible to be used to predict dust concentration changes in different work faces under different climate conditions and to provide a scientific base for pollution control. This study provides several practical solutions where the prediction of dust concentrations at designated work areas will allow construction companies early warning to implement mitigation measures before it becomes a serious health hazard. In addition, it provides an opportunity to re-evaluate those hazardous work in the light of these revelations. The outcome of this study is both original and useful for both construction companies and regulatory agencies. It can better predict the concentration of construction dust in different operating areas and different weather conditions and provide a guide for the prevention and control of construction dust.

## 1. Introduction

With the rapid economic development, China's construction industry has been ushered into a period of large-scale construction and infrastructure development. The increasing scale of construction activities, including building construction, building demolition, equipment installation, and so on, has aggravated particle pollution. The majority of construction activities are in-situ and organized in the open air. In addition, material transportation, loading and unloading, and stockpiles of earthwork cause large-scale unavoidable emissions. The large particles of these emissions tend to settle down near the construction site after being raised. However, small particles tend to flow with the wind and enter the atmosphere to form suspended solids that are commonly known as construction dust [1]. According to past research, construction dust has been considered to be an important source of particle pollution [2–4].

The formation of construction dust has been widely studied by scholars for years, which included dust monitoring technologies [5], dust emission factors [6, 7], dust diffusion rules [8–10], dust pollution characteristics [11, 12], health hazard evaluation [13–15], and dust prevention and control measures [16]. So far, instrument sampling has been the most commonly used method to monitor construction dust particle concentration. Gao [17] measured the TSP (total suspended particulate) concentration using the HXF-35 dust sampler and the TSP as a monitoring index of construction dust. In response to the complexity and uniqueness of construction sites, Ma [18] adopted unmanned aerial vehicles and image recognition technologies to design an automatic monitoring system of construction dust pollution sources and analyzed construction dust from three aspects: tests for construction dust pollution sources, identification of construction dust polluted areas, and characteristic comparison of construction dust pollution sources.

In order to quantify dust data acquired from monitoring, researchers have employed construction dust emission factors and emissions via three commonly used research methods [19]: exposure profiling method, four-dimensional Flux model, and Flux-FDM method. Tian [20] built a mathematical model, a four-dimensional flux model, which is similar to the exposure profiling method proposed by the U.S. Environmental Protection Agency, and a set of construction dust emission monitoring plan matched with this model. The model also combined the actually measured data of more than 40 construction sites for a quantitative assessment of emissions and emission factors of construction dust. After analyzing relevant data of a Tianjin building construction site, Zhao [21] set up the Flux-FDM model, which is used for the estimation of PM10 emissions of construction, and combined the dust emission factors and construction dust influencing factors obtained through the nonlinear fitting. They found wind velocity and the superficial dust water content are key factors that affect dust emissions. However, construction dust emission is not only subjected to the influence of climate factors, but also to the monitoring height, construction intensity, and other factors.

In the studies of emission characteristics of construction dust particles, Tian [22] studied the vertical and horizontal diffusion laws of construction dust at the boundary of the construction site by monitoring the change of the dust fall concentration near the construction site. They found construction dust fall concentration is inversely proportional to the square of height on the same plane of the construction site boundary. The same correlation was also observed between the dust concentration and the square of the distance from the monitoring point to the center on the same height. Li [23] chose typical residential construction projects in Beijing and set up dust concentration collection points in major work areas during three different periods, namely earthworks, frame, and partitions and interior decoration. They conducted on-site monitoring with TSP as the monitoring index. By comparing the dust pollution status of different construction activities, Li [24] analyzed dust emission characteristics and major distribution principles, and the results suggested that dust emissions of different construction activities significantly differed from each other in terms of their concentration, which, to be specific, showed that the dust concentration during the construction of frame is lower than that of the earthworks. The emission intensity of the former is more stable, and the overall dust concentration of the partitioning and interior decoration stage was high but stable. Hou [25] selected the construction sites in Mentougou District and Daxing District of Beijing as the monitoring objects and used the light scattering method and the gravimetric method to measure the dust at different points of the construction site. The results suggested that the dust concentration distribution characteristics are different in different areas of the earthwork construction site. The dust concentration of the foundation pit is much higher than that of the main entrance and downwind area, and the construction site with poor dust prevention level is more likely to produce high-concentration dust pollution.

To sum up, researchers have comprehensively studied dust emissions characteristics of construction work, but most researches have focused on the analysis of the overall dust emission levels and characteristics of the entire construction site. Nevertheless, research into dust emissions characteristics of different work phases of construction is still lacking. The construction process is not homogenous and hence different work phases generate different dust concentrations, dust types, and hazards. A building's construction process goes through three distinctly unique stages: foundation, frame, and internal partitions/finishes. Different from the first two stages, the third mainly happens indoor. Therefore, dust generated during the third stage would not influence the external environment, and the dust characteristics are significantly different from those of the other two stages. Meanwhile, researchers have also done a lot of work in the prediction of construction dust particles. In the establishment of construction dust prediction models, researchers mostly use traditional multiple linear regression models [26, 27], but they have great limitations and cannot capture the relationship between the concentration of dust emission particles and dust monitoring factors, resulting in predictions are not accurate. While a back propagation (BP) neural network can overcome this limitation very well, it can build a very complex nonlinear model, which can well reflect the nonlinear relationship between particle concentration and dust monitoring factors [28].

Hence, in order to accurately portray the dust emission concentration of outdoor construction, this article mainly focuses on the first two stages, namely foundation and construction of the frame of a building. Based on the field data monitoring, dust emission characteristics of these two stages are studied, and dust emission characteristics under multiple pollution sources are analyzed. Meanwhile, a BP neural network model is built using the monitored data. This model is employed to perform simulation analysis of dust emission concentrations in different work areas and predict construction dust concentrations under different conditions.

## 2. Division of Work Areas and Layout of Monitoring Points

The foundation and construction of frames are the main stages of a building construction, whose activities are quite different. Foundation work mainly includes preparation of site, excavation, slope support, filling disposal, rebar processing, concreting, etc. Among them, excavation, slope support, and filling would form part of the foundation excavation. Therefore, the foundation excavation area is chosen as a monitoring point. Activities in the rebar processing area and concreting area differ from each other significantly; therefore, they should be two separate monitoring points. The construction of the frame mainly covers formwork, bar bending, rebar processing, concrete mixing, concrete pouring, and timberwork. Additionally, activities such as formwork demolition and setup, floor rebar binding, concrete pouring, and scaffolding are all done on the construction floor or nearby. Since these work areas are close to each other and have a similar construction environment, the floor work area is set up as a monitoring point. Meanwhile, the rebar processing area, concrete mixing area, and timberwork area are set up as other monitoring points. Moreover, vehicles transporting construction materials during these two stages can easily raise road dust. Therefore, the road area where the vehicles travel in and out of the site was set up as a monitoring point. The profile of all monitoring points in this research is presented in Table 1.

As shown in Table 1, there are 9 monitoring points set up for the two construction stages. The type of dust in different work areas varies different, which primarily includes silicious dust, cement dust, and timber dust. As the most commonly seen dust type, silicious dust generally comes from the soil, which is diffused into the air through natural wind and by vehicles. Cement dust is generally caused by the dust settlement during the loading and unloading of cement bags, transportation process, and dust diffusion during the feeding process, which is common in the concrete mixing area. Timber dust refers to the dust generated during the erection of timber formwork [29].

## 3. Construction Dust Monitoring

### 3.1. Monitoring Index, Equipment, and Methods

At present, there are four main monitoring indicators to measure construction dust, namely dust fall, TSP, PM10, and PM2.5. The total suspended particle (TSP) is defined as the suspended particle whose aerodynamic diameter is smaller than 100 *μm*, and from a particle size perspective, TSP includes particulate matter 10 (PM10) [30]. The increasing mass concentration of TSP in the air can increase the morbidity of chronic obstructive pulmonary diseases, cardiovascular diseases, cerebrovascular diseases, and acute respiratory tract infections [31, 32]. Compared with direct monitoring of PM10 concentrations, monitoring TSP concentrations is less expensive and simpler to operate and can increase the density of monitoring sites and enable larger data collections [33]. Therefore, considering the scientificity and operability of monitoring indicators, combined with the consideration of construction site conditions and dust monitoring costs, the TSP concentration in the air is chosen as the construction dust monitoring indicator. In this research, the TSP concentration is monitored using the dust sampler HXF-35. Measurement results of this instrument can accurately reflect the position and occurrence time of dust pollution and realize multipoint simultaneous monitoring to acquire mass data.

This research refers to the Chinese national standard, “Determination of Dust in the Air of Workplace–Part 1: Total Dust Concentration” and uses the filter membrane increment method for measurement. Before sampling, the filter membrane is weighed. During the process of sampling, the dust sampler HXF-35 is installed on an A-frame holder. Under the obligation of not influencing the construction operations, the sampling point can be kept as close to the operator as practically possible, and the sampling flow rate is set to be 20 L/min. After the end of sampling, all samples are taken back to the lab for weighing and data recording. The TSP concentration can be given by the following equation:

(1) $$\begin{array}{c} c=\frac{m_{2}-m_{1}}{V*t}*1000, \end{array}$$

where *c* denotes the total dust concentration (mg/m^3^), *m*_2_ denotes the membrane quality after sampling (mg), *m*_1_ denotes the membrane quality before sampling (mg), V denotes the sampling flow (mg), and *t* denotes the sampling time (min).

Because of sharp differences in dust concentration at different monitoring points, the monitoring points should be selected according to the practical situations. If there is no serious dust within the vicinity, the sampling time should be above 60 min. If the monitoring point is severely affected by pollution, the sampling time should be controlled within 30 min. The dust concentration of every monitoring point should be monitored for at least four different periods of a day to ensure the completeness and accuracy of dust data. In addition to dust monitoring of different work areas, meteorological data should be recorded, including, temperature, wind velocity, and humidity.

### 3.2. Overview of the Project Monitored

**Figure 1:**
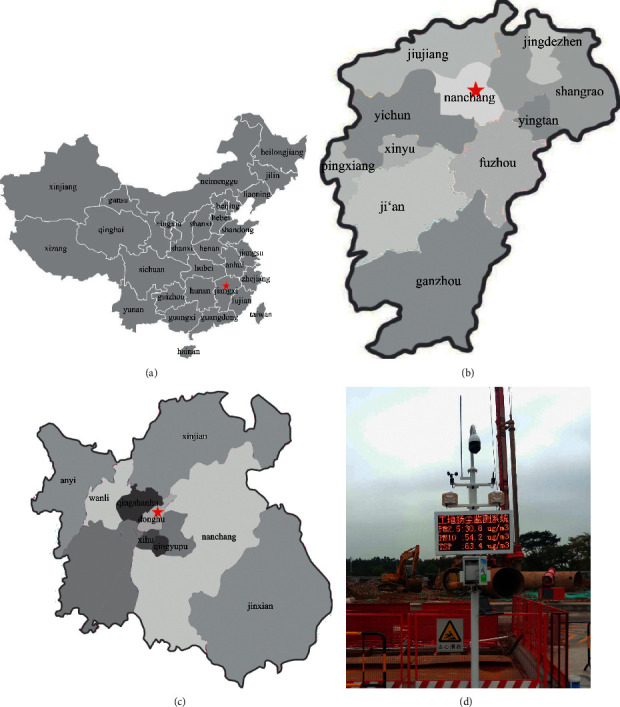
Location of the project.

This research chose a residential construction project in the Donghu District of Nanchang, as shown in Figure 1. Nanchang is located at 115°27′ -116°35′ *E* and 28°10′ - 29°11′ S, which is characterized by a moist monsoon climate of the mid-subtropical region with a pleasant temperature and ample sunlight. The average annual temperature of Nanchang is between 17°C and 18°C, and its average annual precipitation is around 1,600 mm. The meteorological conditions in Nanchang are characterized by a high frequency of calm wind, a high frequency of atmospheric stability, and a high frequency of near-earth inversion layers. The frequencies of calm winds in the four seasons are 25.9%, 24.8%, 21.4%, and 26.6%, respectively. During the calm wind period, the wind speed is small, about grades 1-2, and the temperature inversion phenomenon lasts for a long time, which inhibits the diffusion and dilution of atmospheric pollutants in Nanchang. According to relevant researches, calm wind and temperature inversion are the most important meteorological conditions that cause serious air pollution [34].

## 4. Dust Concentration Emission Characteristics

### 4.1. Dust Concentration Monitoring Result

According to the “Occupational Exposure Limit for Hazardous Agents in the Work-place,” it can be seen that the standard limit of dust concentrations is related to dust types. In order to compare the dust emission concentration of different work areas, this article chose the standard limit for different types of dust concentration to calculate the average concentration, excess multiple, measurement point yield, and other indices, as shown in Table 2.

As shown in Table 2, the average construction dust concentration of the foundation during excavation is 0.988, which is the lowest of value and falls within the allowable limit of the silicious dust. There are two reasons for this. Firstly, the soil water content in the construction area is high, which retards the formation of dust. Secondly, earthwork is mainly carried out by large machinery, whose tracks can help consolidate the soil beneath, preventing the generation of dust. The dust emissions of the road area and the concrete mixing area are severe. The average dust concentration, dust concentration peak, and sample variance of the concrete mixing area are 7.392, 17.760, and 10.017, respectively, which are considerably higher than those of other work areas. The average dust concentration of the road area is 4.287, which is around four times the average dust concentration of the foundation area and rebar processing area. This suggests that concrete processing and vehicular traffic are the main sources of dust during the foundation work.

During the construction of the frame, the average dust concentration of timber formwork area is 8.697, which is around eight times the average of the floor area and exceeds the average of the concrete mixing area and the road area by four-folds. The measurement point yield of the work area is just 12%, meaning that the dust emissions are severe in the timber formwork area and also constitute the main dust source of this stage. This is mainly caused by a tight workspace, which slows down dust diffusion. Emissions from the road area are the second largest for this stage, whose average dust concentration, measurement point yield, and sample variance are all below those of the timber formwork area. The average dust concentration of the concrete mixing area is below that of the floor work area and the rebar processing area. The dust concentration of the concrete mixing area is within the allowable standard, its exceeding multiple is 0, and its measurement point yield is 100%. All these data suggest that dust emissions of the concrete mixing area are slightly lower than those of the floor area and the rebar processing area. Compared with the floor work area, the rebar processing area has a higher average concentration, exceeding multiple, and measurement point yield, implying that dust emissions of the latter are more severe than those of the former; because the dust pollution in the vertical direction of the spread is limited, the dust concentration of the floor area decreases constantly with the increase of floors.

### 4.2. Comparison of Construction Dust in the Same Work Area but at Different Stages

Table 3 summarizes the average construction dust concentration, average exceeding multiple, and variation of the indices during earthwork and structural frame stages. Overall, with the exception of the rebar processing area, a decrease in the average construction dust concentration and average exceeding multiple could be observed, when construction activities move from the foundation to the structural frame.

Based on average concentration, emissions in the rebar processing area are on an upward trend, because of the heavy demand placed on rebar for the structural frame. Though road area is the major dust source during foundation, it decreases by more than 50% when construction moves to the structural frame. There are two main reasons for the decline. Firstly, there is more bare soil during the earthwork as most pavements have not been hardened. However, by the time construction moves over to the structural frame, most of these road surfaces are hardened, reducing dust raised by vehicles. Secondly, during the structural frame, there are fewer vehicles transporting soil from and to the site. The dust concentration of concrete mixing during earthworks is three times higher than that of structural frame. This is mainly because concrete is transported using a pump for pouring into the formwork, whereas for foundation, a pump is not used. The cement, before entering the compression pump, has full contact and reaction with water and aggregates, thus evading the generation of cement dust.

From the average exceeding multiple perspective, vehicle movements cause severe dust during both foundation and structural frame stages. As to dust emissions of the rebar processing area and the road area, their average exceeding multiple varies significantly and drops by a large margin. In particular, the average exceeding multiple of the concrete mixing area during the structural frame has dropped to zero.

### 4.3. Comparison of Work Areas with Major Construction Dust Emissions

Table 4 summarizes work areas with severe construction dust emissions in the two stages. During foundation work, road area, concrete mixing area, and rebar processing area are the main dust-generating areas. However, during the construction of structural frame, timber formwork area, road area, and rebar processing areas have emerged as major dust-generating areas.

**Figure 2:**
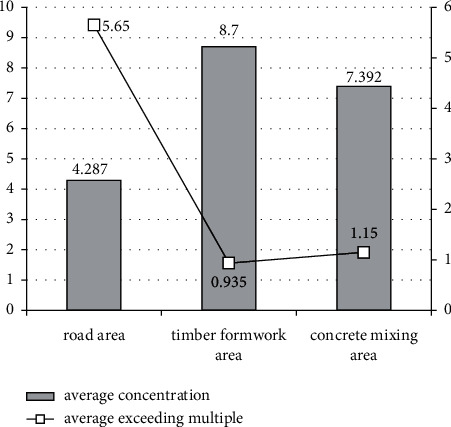
Comparison of dust concentration and excess multiple in work areas with severe emissions.

A comparison of average dust concentration and average exceeding multiple of the three work areas is shown in Figure 2. The highest average excess multiple is found for the road area, which is five times as high as that of the other two. Hence, these three work areas should be the key areas for construction dust prevention and control during construction.

## 5. Establishment of Dust Concentration Prediction Model Based on the BP Neural Network

Considering the complexity of building construction process, multiple work phases, high emission randomness, and difficulty of quantifying dust pollution, it is very important to model dust concentrations at different work phases. Therefore, this research conducts a simulation prediction of dust particle concentration at different work areas in an attempt to build a construction dust particle concentration prediction model using the BP neural network.

### 5.1. Overview of the BP Neural Network

**Figure 3:**
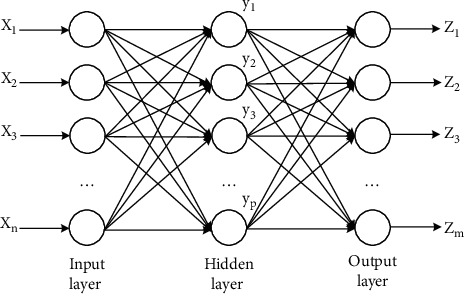
BP neural network structure.

As one of the most widely used models, the BP neural network has found applications in many fields [36–38]. The BP neural network is defined as a feedforward neural network or backpropagation neural network, which is characterized by the forward propagation of signals and backward propagation of errors. Generally speaking, the BP neural network consists of the input layer, hidden layer, and output layer [39], and its structure is presented in Figure 3. The optimization capacity of the BP neural network has a close bearing on its structure—a structure characterized by variability, nonlinearity, error tolerance, self-adaption, and autonomous learning.

### 5.2. Dust Concentration Prediction Model

The neural network model adopted in this research to build the construction dust concentration prediction model features a three-layer network structure, in which there are three neurons in the input layer, namely the temperature, moisture, and wind velocity [40]. The initial hidden layer has 20 neurons, and the output layer has one neuron that is dust concentration. In other words, it is a model with a 3-20-1 three-layer network structure. Meanwhile, MATLAB2016a is adopted as the numerical computing platform. Before prediction, the BP neural network should first receive network model training to get equipped with the ability of memorization and prediction. To the end, the “Rand” function is used to acquire 60 samples from every monitoring point, and the first 50 samples are adopted as the training set, while the other 10 as the test set. The iteration items are set as 100, and the learning rate is taken as 0.01.

### 5.3. Dust Concentration Simulation of Different Work Areas

**Figure 4:**
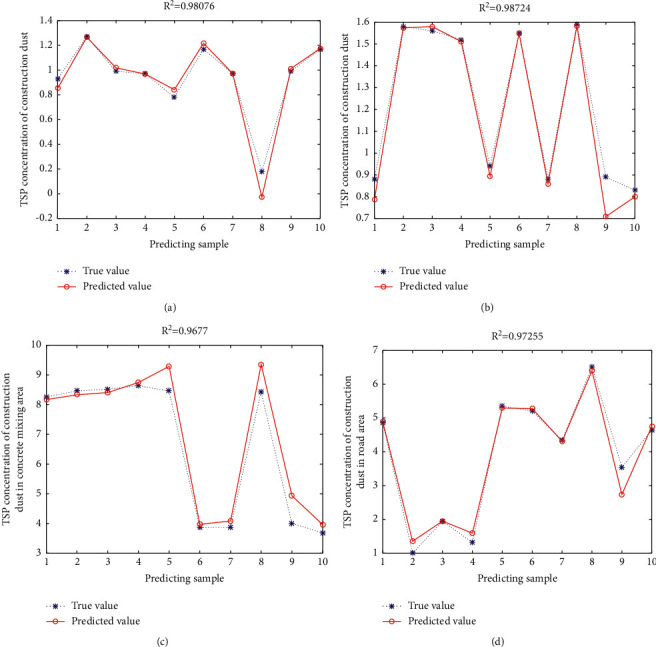
Comparison between the predicted values and the measured values of dust particle concentration during the foundation stage: (a) foundation excavation area, (b) rebar processing area, (c) concrete mixing area, and (d) Road area.

The model with a 3-20-1 network structure thus built is used to conduct simulated prediction of various work areas.  Figure 4 shows the actually measured values and predicted values of the dust emission concentration of different work areas of the foundation stage.  Table 5 shows the correlation coefficient of simulation prediction of different work areas during the foundation and structural frame stages. Hence, R2 below denotes the decision coefficient of regression analysis, which is 0.9807, 0.98724, 0.9677, and 0.97255 in the foundation area, rebar processing area, concrete rebar area, and road area of the foundation stage, respectively. The prediction results and the actually measured value show a high degree of fitting, indicating favorable simulation prediction effects as shown in Figure 4. From Table 5, it can be seen that R2 of the floor work area, concrete mixing area, rebar processing area, timber formwork area, and road area are 0.9749, 0.9097, 0.9556, 0.9608, and 0.9988, respectively. Combining the results of Table 4 with the results of Figure 4, the neural network training results of different work areas in the structural frame stage are favorable.

**Figure 5:**
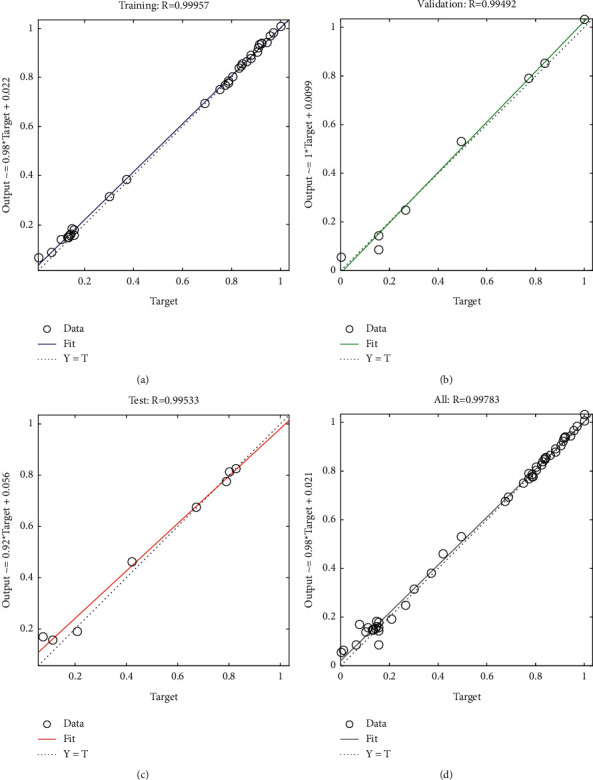
Regression analysis results of the road area during the structural frame stage: (a) training: *R* = 0.99957, (b) validation: *R* = 0.99492, (c) test: *R* = 0.99533, and (d) all: *R* = 0.99783.

Table 6 shows the regression results of the predicted output and the target data of different work areas during the two stages.  Figure 5 shows the regression analysis results of the predicted output and the target data of the road area during the structural frame stage, where training, validation, test, and all represent the regression coefficient *R* of the training samples, verification samples, test samples, and integrated samples, respectively. The regression coefficients are above 90% as shown in Table 6 and are generally close to 1. By combining the results demonstrated in Figure 5, it is concluded that the model generates favorable simulation prediction results. It also shows the feasibility of developing a construction dust emission concentration prediction model based on the BP neural network.

## 6. Discussion

Research results suggest that, during the construction period, dust emissions of different work areas differ from each other significantly, which aligns with the previous research findings [41]. However, numerical simulations of construction dust at present are mostly based on the gas-solid two-phase flow theory to simulate the diffusion based on rules of the wind velocity, height of the generation source, and dust concentration [42, 43]. It is not based on the characteristics of on-site construction activities and their influences on dust emissions. This is a major gap in the extant literature, and hence some of the highly polluting areas are neglected in auditing and monitoring schemes. In order to fill the above gap in the knowledge, this research chose the wind velocity, temperature, and moisture as input factors to build a construction dust simulation prediction model for different work areas of a construction site, focusing primarily on two work stages that happen in the open air. Hence, the model considers not only the influence of the meteorological conditions, but also the influence of construction activities on dust emissions. The regression coefficients show that the predicted values and the measured values demonstrate good agreement. Therefore, the established model should be capable of well-predicting construction dust concentration changes in different work areas and under different weather conditions and providing a scientific base for its control, diffusion, and pollution.

In order to verify the validity of the construction dust prediction model, ten test samples are randomly chosen from the road area during the construction of the structural frame for prediction as shown in Table 7. The relative error between the predicted values and the actual values of the ten samples are all around 0.01, meaning that the construction dust prediction model proposed by this article can obtain favorable results. In addition, the neural network construction dust prediction model is easy to use and well demonstrate the prediction results in the form of curves. The prediction of dust concentrations in different work areas of a construction site has several positive implications. It allows the construction company to monitor dust levels at different stages of work and plan strategic interventions before it is too late. It could prevent dust-related long-term illnesses among workers who are routinely involved in such tasks.

For example, an investigation by the Australian Broadcasting Cooperation (ABC, 2019) revealed that the workers who are involved in installing stone kitchen bench-tops have reported silicosis due to exposure to silica dust (http://www.aap.com.au/,2019). The report states “doctors are worried Australia is facing the worst occupational lung disease crisis since the peak of the asbestos disaster” as number of stonemasons in New South Wales, Victoria, Australian Capital Territory, and Queensland have been reported to the hospital with accelerated silicosis of alarming levels. However, the builders involved in residential construction in Australia have never been on the spotlight for any violation of WHS laws related to dust pollution. The reason is, construction dust is considered as “nuisance dust” because most auditing and monitoring (if all happens) is based on the overall measurement results rather than on designated areas. Based on these overall measurements, the site does not exceed the regulatory thresholds set by the Environmental Project Agency (EPA) of Australia. However, if measurements were taken at different work areas, some areas could be well above those limits. Therefore, the prediction of dust concentrations at designated work areas should be a high priority for construction companies. Furthermore, regulatory agencies should avoid blanket rulings on construction sites as different activities have varying dust generation potential, some of which could be very harmful to workers and the neighboring community.

## 7. Conclusion

Due to the complexity, continuity, and time-varying characteristics of construction dust emissions, traditional regression prediction models cannot accurately predict the concentration of the dust emissions. Therefore, in order to simulate construction dust under multiple pollution sources, this article monitored dust emissions during two important stages of a residential construction project, namely foundation and structural frame. The study identified training and learning samples for the neural network, compiled the learning and training algorithm, and built the neural network model reflecting dust emission concentrations in different work areas. Based on the results of simulation prediction, the output data of the BP neural network model demonstrate a favorable and ideal correlation, and compared with the traditional regression model, the dust concentration prediction model established by the BP neural network is feasible to be used to predict dust concentration changes in different work areas and under different climate conditions, which can provide a scientific base for pollution control.

The majority of work areas have exceeded the allowable upper limit of TSP concentration stipulated by relevant standards. This means that most work areas are suffering from the serious concentration of dust pollution under multiple pollution sources. The dust emission differences of work areas are pronounced. To be specific, the areas with serious dust emission concentrations during foundation include the road area, concrete mixing area, and rebar processing area. The areas with serious dust emission concentrations during the construction of structural frame include the timber formwork area, road area, and rebar processing area. While the road area in foundation construction, concrete mixing area, and wood formwork area in structure construction are the key areas of dust emission in construction period.

The prediction of dust concentrations in designated work areas will provide construction companies with an early warning to implement mitigation measures before it becomes a serious health hazard. In addition, it provides an opportunity to re-evaluate those hazardous work in the light of these revelations. Although this study showed that the BP neural network could develop such early warning, the study only used two stages of a long and laborious construction process as a demonstration. Further research is needed in other stages and activities to evaluate the true potential of BP neural network simulations and to demonstrate their suitability. Further research into other stages of construction could reveal activities that are prone to severe dust emissions which at present are not considered as hazardous by builders or regulatory bodies.

## Figures and Tables

**Table 1:** Profile of dust monitoring points.

Construction stage	Monitoring points	Construction dust types	Major activities
Foundation	Foundation excavation area	Silicious dust	Soil excavation, slope support, earthwork compaction
	Rebar processing area	Silicious dust	Rebar transportation, processing, and storage
	Concrete mixing area	Cement dust	Concrete mixing and transportation
	Road area	Silicious dust	Earthworks and construction material transportation

Structural frame	Floor area	Silicious dust	Rebar binding, erection of formwork, scaffolding work, and demolition
	Concrete mixing area	Cement dust	Concrete mixing and transportation
	Rebar processing area	Silicious dust	Rebar transportation, processing, and storage
	Timber formwork area	Timber dust	Timber formwork and other timber processing
	Road area	Silicious dust	Transportation of premixed concrete and other construction materials

**Table 2:** Concentrations of dust at different monitoring points of work areas.

Construction periods	Dust monitoring points	Dust types	Average concentration (mg/m^3^)	Scope of excess multiple	Measurement point yield (%)	Variance
Foundation	Foundation excavation area	Silicious dust *a*	0.988	0.000–0.320	72.000	0.042
	Rebar processing area	Silicious dust *a*	1.103	0.000–0.590	70.000	0.082
	Concrete mixing area	Cement dust *b*	7.392	0.000–3.440	37.000	10.017
	Road area	Silicious dust *a*	4.287	0.000–5.650	13.000	3.391

Structural frame	Floor area	Siliciousdust *a*	1.148	0.000–0.600	60.000	0.066
	Concrete mixing area	Cement dust *b*	2.093	—	100.000	0.791
	Rebar processing area	Silicious dust *a*	1.374	0.000–0.740	38.000	0.127
	Timber formwork area	Timber dust *c*	8.697	0.000–3.740	12.000	4.855
	Road area	Silicious dust *a*	2.124	0.000–2.200	30.000	0.860

Note. The concentration of silicious dust, cement dust, and timber dust is *a*, PC-PWA = 1 mg/m^3^, *b*, PC-PWA = 4 mg/m^3^, and *c*, PC-PWA = 3 mg/m^3^ [35], respectively.

**Table 3:** Comparison of construction dust in the same work area at different stages of construction.

Monitoring point	Average concentration (mg/m^3^)			Average exceeding multiple		
	Foundation	Structural frame	Variation	Foundation	Structuralframe	Variation
Rebar processing area	0.988	1.374	39.070	0.590	0.740	25.420
Concrete mixing area	7.392	2.089	−71.730	3.440	0.000	−100.000
Road area	4.287	2.124	−50.450	5.650	2.200	−61.060

**Table 4:** Comparison of work areas with severe dust emissions.

Serial number	Foundation	Structural frame
1	Road area	Timber formwork area
2	Concrete mixing area	Road area
3	Rebar processing area	Rebar processing area

**Table 5:** Dust particle concentration of different work areas.

Construction periods	Work areas	*R*2
Foundation	Foundation excavation area	0.9807
	Rebar processing area	0.9872
	Concrete mixing area	0.9677
	Road area	0.9726

Structural frame	Floor area	0.9749
	Concrete mixing area	0.9097
	Rebar processing area	0.9556
	Timber formwork area	0.9608
	Road area	0.9988

**Table 6:** Regression analysis results of the predicted output and the target data of different work areas.

Construction stage	Work areas	Training	Validation	Test	All
Foundation	Foundation excavation area	0.9937	0.9459	0.9833	0.9859
	Rebar processing area	0.9968	0.9135	0.9583	0.9746
	Concrete mixing area	0.9821	0.869	0.9098	0.9586
	Both sides of road	0.9986	0.9913	0.9966	0.9973

Structural frame	Floor area	0.9917	0.9777	0.9908	0.9837
	Concrete mixing area	0.9986	0.9894	0.9988	0.9962
	Rebar processing area	0.9962	0.9956	0.9721	0.9921
	Timber formwork area	0.9968	0.7586	0.9442	0.9885
	Both sides of road	0.9996	0.9949	0.9953	0.9978

**Table 7:** Predicted results of the road area during the structural frame.

Index	1	2	3	4	5	6	7	8	9	10
Measured value	2.65	2.58	2.98	0.87	2.98	3.18	0.95	3.20	3.15	2.65
Predicted value	2.64	2.62	3.04	0.93	3.01	3.19	0.99	3.15	3.15	2.64
Relative error (%)	0.32	1.91	1.88	4.27	1.11	0.53	3.99	1.44	0.06	0.32

## Data Availability

The data used to support the findings of this study are available from the corresponding author upon request.
